# Acute pancreatitis and COVID-19: a new target for infection?

**DOI:** 10.31744/einstein_journal/2022RW6667

**Published:** 2022-02-03

**Authors:** Júlia Aith Balthazar, Ethel Zimberg Chehter

**Affiliations:** 1 Centro Universitário FMABC Santo André SP Brazil Centro Universitário FMABC, Santo André, SP, Brazil.

**Keywords:** Pancreatitis, Coronavirus infections, COVID-19, SARS-CoV-2, Betacoronavirus

## Abstract

This review aimed to investigate whether SARS-CoV-2 is capable of infecting the gland and causing acute pancreatitis, and the peculiarities in the management of these cases. The research was conducted through PubMed^®^ database, and 62 articles were systematically selected for analysis. Differences were found in the literature; however, there are important warnings, such as the presence of hyperlipasemia, clinical and imaging findings suggestive of acute pancreatitis in the presence and even in the absence of respiratory symptoms. Attention should be paid to clinical and imaging findings during this virus infection, since it is possible to identify these two diseases early. Therefore, it is possible to detect and isolate these patients more quickly, providing the correct care and decreasing the morbidity and mortality of two potentially severe diseases.

## INTRODUCTION

Already in its third wave of new cases, severe acute respiratory syndrome coronavirus 2 (SARS-CoV-2) has continued for one year keeping the world under a pandemic with more than 2.8 million deaths.^(1)^ The virus was initially detected in December 2019 in Wuhan, China, with atypical findings of viral pneumonia. On March 11, 2020, the virus had spread across the globe, and a pandemic for coronavirus 2019 disease (COVID-19) was determined by the World Health Organization (WHO).^(2)^

As the number of cases increased, extrapulmonary symptoms were found, such as ageusia, anosmia, diarrhea, myocarditis, and urticaria, among others.^(3)^ This was due to the ability of the virus to infect the host cells through the angiotensin-converting enzyme 2 (ACE2) receptor, which is present in various tissues, such as the respiratory tract, cardiovascular system, kidneys, and intestines.^(4)^ Among the gastrointestinal manifestations, an incidence of 3% to 79% of symptoms was detected, ranging from vomiting, anorexia, diarrhea, and nausea to gastrointestinal bleeding.^(5)^

Miao et al., reported the first case of acute pancreatitis in a patient with COVID-19 without respiratory symptoms.^(6)^ Along the same line, other studies were published regarding pancreatic involvement by SARS-CoV-2, suggesting that the virus can cause pancreatic injury.^(7)^ Supporting these findings, Liu et al., identified that ACE2 receptor is expressed in the pancreas in healthy people, and is even slightly more significant in pancreatic tissue than in the lungs, proposing that SARS-CoV-2 may cause pancreatic injury by binding to pancreatic ACE2.^(8)^

Few pathological studies in patients with COVID-19 have been performed with emphasis on the pancreas to support these hypotheses, but it was possible to find autopsies in the literature describing degeneration of pancreatic cells,^(9)^ interstitial fibrosis, and lipomatosis separating the acinar cells^(10)^ in these patients, besides detecting SARS-CoV-2 RNA in pancreatic cells.^(11)^

Acute pancreatitis is a potentially severe disease, with the main causes being cholelithiasis (40% to 70%), and alcoholism (25% to 35%). Other rare causes (10%), such as medications, trauma, endoscopic retrograde cholangiopancreatography (ERCP), hyperkalemia, hypertriglyceridemia (>1,000mg/dL), infection, genetics, and autoimmune diseases.^(12)^ Among the infectious agents are viruses (hepatotropic, coxsackievirus, cytomegalovirus (CMV), human immunodeficiency virus (HIV), herpes simplex virus, paramyxovirus, and varicella-zoster virus), bacteria (mycoplasma, *Legionella, Salmonella*, and leptospira), fungi (*Aspergillus*), and parasites (toxoplasma, *Cryptosporidium,* and *Ascaris*).^(13)^

In the current context, in which several articles described acute pancreatitis in patients who tested positive for COVID-19 infection, it is suggested the virus may be another infectious cause of pancreatic injury, as well as several other agents described here.

## OBJECTIVE

To investigate the capacity of SARS-CoV-2 of causing acute pancreatitis and the peculiarities in diagnosis and care of these cases.

## METHODS

**Figure 1:**
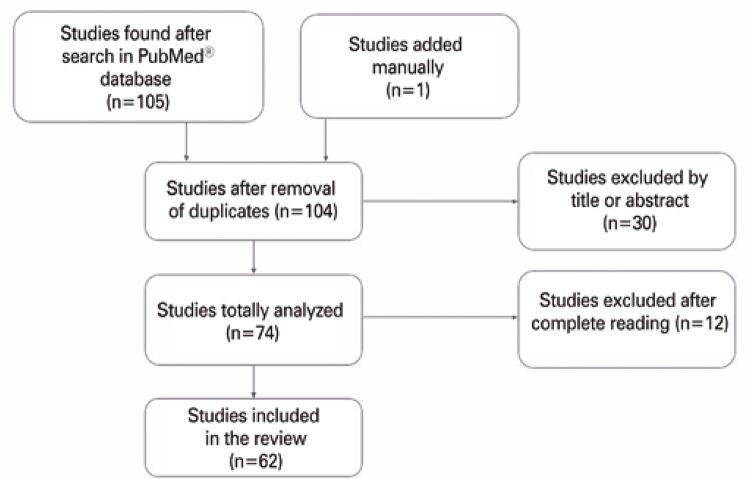
Preferred Reporting Items for Systematic Reviews and Meta-Analyses (PRISMA) diagram

The database chosen was PubMed^®^. On February 16, 2020, articles with the words “COVID-19” and “acute pancreatitis” were selected. From this search, 105 articles were found; one article was added manually. Inclusion criteria were studies that associated acute pancreatitis with COVID-19, as well as those about hyperlipasemia or hyperamylasemia and COVID-19. Exclusion criteria were articles about acute pancreatitis from another well-defined cause; in letter or comment form; with patients with a negative result for COVID-19; about the treatment of pancreatitis; about multisystem inflammatory syndrome of the child; and that did not deal with acute pancreatitis. Figure 1 shows the Preferred Reporting Items for Systematic Reviews and Meta-Analyses (PRISMA) diagram, based on the reading of titles and abstracts; 30 articles were excluded, leaving 74. Of these, 12 were excluded because they dealt with the mechanism of action of the virus, acute pancreatitis with negative COVID-19 results; they either did not mention the disease or pancreatic alterations, or had a defined cause for acute pancreatitis. Sixty-two articles were then included. The initial analysis was done by the main author and later reviewed by the supervisor of this work. Information such as patients’ age, comorbidities, and personal history, chief complaint, gastrointestinal symptoms, pneumonia, or severe acute respiratory syndrome (SARS), type of COVID-19 diagnostic test, medications, laboratory and imaging tests, and diagnostic criteria for pancreatitis, whenever cited, were used for analysis in this study. Regarding the diagnostic criteria, the Revised Atlanta Classification of acute pancreatitis, of 2012, was considered standard. Among the radiological findings, localized or diffuse enlargement of the pancreas, blurring of peripancreatic fat, presence of periglandular collections, pancreatic necrosis, and pancreatic pseudocyst were considered late presentations. Regarding the increase in pancreatic enzymes, hyperamylasemia shows high sensitivity and low specificity, whereas hyperlipasemia shows high sensitivity and specificity for the diagnosis of acute pancreatitis.

## RESULTS

The research findings were divided into three tables, one containing literature reviews, prospective and retrospective studies (Table 1), another with the case reports (Table 2), and the last with the frequencies of the case report findings (Table 3).

**Table 1:** Data from prospective, retrospective studies, and literature reviews

Studies	Type of article	Patients (n)	Age (median)	Amylase/lipase (U/L)	Diagnosis of pancreatitis	Relation between COVID-19 and acute pancreatitis?
Zippi et al.^(7)^	Review	14	-	-	-	Yes
Suchman et al.^(14)^	Cohort	13	12	Amylase and lipase >3x	INSPPIRE	Yes
Hegyi et al.^(15)^	Meta-analysis	12	-	-	-	Yes
Akarsu et al.^(16)^	Prospective study	316	54	Amylase and lipase >3x	Modified Atlanta Classification	Yes
Gupta^(17)^	Meta-analysis	4	53	Amylase and lipase >3x	-	Yes
Juhász et al.^(18)^	Systematic review	8	-	-	Atlanta Classification	No
McNabb-Baltar et al.^(19)^	Cohort	71	64.9	Lipase >60	Atlanta Classification	No
Miró et al.^(20)^	Case-control	54	-	Amylase >3x	Atlanta Classification	No
Lax et al.^(21)^	Prospective study (autopsy)	11	80.5	-	-	Yes
Rasch et al.^(22)^	Cohort	38	68.5	Mean lipase of 422U/L	Modified Atlanta Classification	No
Inamdar et al.^(23)^	Cohort	32	53.44±16.60	Amylase and lipase >3x	Atlanta Classification	Yes
Dirweesh et al.^(24)^	Cohort	14	55.2±14.8	-	Atlanta Classification	Yes
Hanley et al.^(25)^	Cohort	8	-	-	Autopsy	Yes
Szatmary et al.^(26)^	Cohort	5	42	Median amylasemia of 149U/L	Atlanta Classification	Yes
Gubatan et al.^(27)^	Cohort	8	55.3±18.7	-	-	No
Akkus et al.^(28)^	Cohort	20	55.5±18.9	Mean lipase of 91U/L	Atlanta Classification	No
Samanta et al.^(29)^	Systematic review	5	-	-	-	No
Shiralkar et al.^(30)^	Cohort	10	45.1±19.6	-	Abdominal CT and MRI	Yes
Pandanaboyana et al.^(31)^	Cohort	149	59.9±17.2	Hyperamylasemia	Modified Atlanta Classification	Yes
Goyal et al.^(32)^	Systematic review	7	-	Hyperlipasemia	-	No
Rathi et al.^(33)^	Cohort	83	-	Median lipase of 391U/L	-	No
McGuinness et al.^(34)^	Cohort	650	57	Lipase >3x	Atlanta Classification	No
Wang et al.^(35)^	Cohort	52	55±15	Median amylase of 115±25U/L Median lipase of 71±34U/L	>90U/L amylase and >70U/L lipase with pancreatic injury	Yes

INSPIRRE: International Study Group of Pediatric Pancreatitis: In Search for a CuRE; CT: computed tomography; MRI: magnetic resonance imaging.

**Table 2:** Key data from the case reports

Article data	Miao et al.^(6)^	Aloysius et al.^(36)^	Anand et al.^(37)^	Hadi et al.^(38)^	Hadi et al.^(38)^	Meireles et al.^(39)^
Age	26	36	59	47	68	36
Sex	Female	Female	Female	Female	Female	Female
Past history	-	Obesity and anxiety disorder	Thrombophilia and prior cholecystectomy	-	HTN, hypothyroidism, and osteoporosis	Post-HELLP syndrome, CKD, and HTN
Initial symptoms	Vomiting, epigastric pain, and fever	Fever, dry cough, dyspnea, nausea, vomiting, and diarrhea	Fever, sore throat, and myalgia	Fever, headache, anorexia, sore throat, and dyspnea	Epigastric pain and fever	Dry cough, fever, and dyspnea
GIT symptoms	Yes	Yes	Yes	No	Yes	Yes
Abdominal pain	Epigastric	Band-like epigastric pain irradiating towards the back	-	-	Epigastric	Band-like epigastric pain
Pneumonia	Yes	Yes	Yes	Yes	Yes	Yes
SARS	No	Yes	No	Yes	Yes	Yes
Test to confirm COVID-19	RT-PCR	RT-PCR	RT-PCR	RT-PCR	RT-PCR	?
Amylase, U/L	Not done	325	Not done	>1,500	934	718
Lipase, U/L	430	627	Not done	Not done	Not done	631
CRP, mg/dL	1.38	1.95	6.27	-	7.7	11.9
Imaging test suggestive of acute pancreatitis	Abdominal CT scan	No	Abdominal CT scan	Abdominal ultrasound	No	Abdominal CT scan
Triglycerides, mg/dL	Not done	136	Not done	Normal	Normal	120
Cholelithiasis or alcoholism	No	No	No	No	No	No
Diagnosis of pancreatitis	Imaging, clinical, and laboratory	Modified Atlanta Classification	Suggestive abdominal CT scan	Modified Glasgow Coma Score for Acute Pancreatitis (5 points)	Modified Glasgow Coma Score for Acute Pancreatitis (5 points)	Imaging, clinical, and laboratory
CUM	-	Alprazolam	-	-	Losartan, levothyroxine, alendronate, and cyanocobalamin (Vitamina B12)	Nifedipine and carvedilol
Medication on admission	-	-	-	-	-	-
Complications		NIV	-	OTI and mechanical ventilation, hemodialysis, and ECMO	OTI and mechanical ventilation, and hemodialysis	-
Outcome	Resolution	Resolution	Resolution	Not available	Not available	Resolution

**Article data**	**Shinohara et al.^(40)^**	**Meyers et al.^(41)^**	**Cheung et al.^(42)^**	**Kandasamy et al.^(43)^**	**Lakshmanan et al.^(44)^**	

Age	58	67	38	45	68	
Sex	Male	Male	Male	Female	Male	
Personal antecedents	HTN	HTN and cholecystectomy	-	?	DM, HTN, and CKD	
Initial symptoms	Fever and dyspnea	Epigastric pain, fever, tachycardia, and tight abdomen	Fever, intense epigastric pain, nausea, and vomiting	Epigastric pain irradiating towards the back, nausea, and vomiting	Loss of appetite and nausea	
GIT symptoms	Yes	Yes	Yes	Yes	Yes	
Abdominal pain	-	Epigastric	Epigastric	Epigastric irradiating towards the back	Denies	
Pneumonia	Yes	Yes	No	Yes	No	
SARS	Yes	No	No	No	No	
**Article data**	**Shinohara et al.^(40)^**	**Meyers et al.^(41)^**	**Cheung et al.^(42)^**	**Kandasamy et al.^(43)^**	**Lakshmanan et al.^(44)^**	
Test to confirm COVID-19	RT-PCR	RT-PCR	RT-PCR	RT-PCR	RT-PCR	
Amylase, U/L	383	Not done	Not done	364	1,030	
Lipase, U/L	Not done	5,295	1,219.2	293	2,035	
CRP, mg/dL	11.51	Not done	Not done	Not done	15.8	
Imaging test suggestive of acute pancreatitis	Abdominal CT scan	Abdominal CT scan	Abdominal CT scan and MRI	Abdominal CT scan	Abdominal CT scan	
Triglycerides, mg/dL	Not done	Normal	Normal	Not done	Normal	
Cholelithiasis or alcoholism	No	Mild alcoholism	No	No	No	
Diagnosis of pancreatitis	Imaging, clinical, and laboratory	Abdominal CT	Abdominal CT	Abdominal CT	Imaging, clinical, and laboratory	
CUM	-	-	-	-	-	
Medication on admission	Piperacillin- tazobactam, azithromycin, favipiravir, nafamostat mesilate, and methylprednisolone	-	-	Empirical antibiotic	Empirical antibiotic	
Complications	OTI and ECMO	Not available	-	NIV	-	
Outcome	Resolution	Not available	Resolution	Resolution	Resolution	

**Article data**	**Gonzalo-Voltas et al.^(45)^**	**Brikman et al.^(46)^**	**Narang et al.^(47)^**	**Wang et al.^(48)^**	**Wang et al.^(48)^**	

Age	76	61	20	42	35	
Sex	Female	Male	Female	Male	Male	
Past history	Hypercholesterolemia and GERD	-	Primigesta, 33- week gestation, obesity, and cholecystectomy	-	-	
Initial symptoms	Epigastric pain and vomiting	Fever, dyspnea, and cough	Dry cough and myalgia	Nausea and epigastic pain	Abdominal pain, nausea, and vomiting	
GIT symptoms	Yes	Yes	Yes	Yes	Yes	
Abdominal pain	Band-like epigastric pain	Diffuse abdominal pain	Epigastric pain irradiating to the back	Epigastric pain irradiating to the back	Epigastric pain irradiating to the back	
Pneumonia	No	Yes	Yes	Yes	Yes	
SARS	No	Yes	Yes	Yes	No	
Test to confirm COVID-19	RT-PCR	RT-PCR	RT-PCR	RT-PCR	RT-PCR	
Amylase, U/L	3,568	142	1,168	132	Normal	
Lipase, U/L	Not done	203	859	382	1,042	
CPR, mg/dL	1.9	-	-	>20	>20	
Imaging test suggestive of acute pancreatitis	Abdominal CT and ultrasound	Abdominal CT	Abdominal CT	-	Abdominal CT	
Triglycerides, mg/dL	Not done	281.67	-	283.44	170.06	
Cholelithiasis or alcoholism	No	No	No	No	No	
Diagnosis of pancreatitis	Imaging, clinical, and laboratory	Modified Atlanta Classification	Presumptive	Ranson	Imaging, clinical, and laboratory	
CUM	Omeprazole 20mg/day	-	-	-	-	

**Article data**	**Gonzalo-Voltas et al.^(45)^**	**Brikman et al.^(46)^**	**Narang et al.^(47)^**	**Wang et al.^(48)^**	**Wang et al.^(48)^**	

Medication on admission	Azithromycin, chloroquine, lopinavir, and ritonavir	Azithromycin, hydroxychloroquine, zinc, tocilizumabe, dexamethasone, enoxaparin, lopinavir- ritonavir, pantoprazole, ciprofloxacin, and clindamycin	-	-	-	
Complications	-	NIV	NIV and premature rupture of membranes, and preterm birth	Cardiac arrest, OTI and mechanical ventilation, and hemodialysis	-	
Outcome	Resolution	Resolution	Resolution	Death	Resolution	

**Article data**	**Tollard et al.^(49)^**	**Acherjya et al.^(50)^**	**Karimzadeh et al.^(51)^**	**Simou et al.^(52)^**	**Mazrouei et al.^(53)^**	

Age	32	57	65	67	24	
Sex	Female	Female	Female	?	Male	
Past history	Morbid obesity and diabetic ketoacidosis	HTN, type 2 DM, breast and larynx cancer, and prior cholecystectomy	HTN and asthma	Type 2 DM, obesity, and prior cholecystectomy	-	
Initial symptoms	Dyspnea, polyuria, polydipsia, and abdominal pain	High fever, myalgia, anosmia, fatigue, and arthralgia	Epigastric pain, nausea, chills, and myalgia. On the 7^th^ day, the patient presented with dyspnea	Fever, dyspnea, myalgia, and arthralgia	Epigastric pain, nausea, vomiting, and mild respiratory symptoms	
GIT symptoms	Yes	Yes	Yes	No	Yes	
Abdominal pain	Abdominal	Epigastric pain irradiating to the back	Epigastric	No	Epigastric	
Pneumonia	Yes	Yes	Yes	Yes	No	
SARS		No	No	No	No	
Test to confirm COVID-19	RT-PCR	RT-PCR	RT-PCR	RT-PCR	RT-PCR	
Amylase, U/L	Not done	Not done	192	-	391	
Lipase, U/L	321	8,352	283	576	578	
CRP, mg/dL	-	25.2	-	4.13	-	
Imaging test suggestive of acute pancreatitis	Abdominal CT scan	Abdominal CT scan	Abdominal CT scan	Abdominal CT scan	Abdominal CT scan	
Triglycerides, mg/dL	Normal	276	80	212.58	Not done	
Cholelithiasis or alcoholism	No	No	No	No	No	
Diagnosis of pancreatitis	Imaging, clinical, and laboratory	Atlanta Classification	Clinical	Balthazar Classification of Abdominal CT	Atlanta Classification	
CUM	-	Radiation therapy, trastuzumab, losartan, metformin, and insulin	-	-	-	
Medication on admission	-	Favipiravir and enoxaparin in prophylactic dose	Levofloxacin, ondansetron, oseltamivir, hydroxychloroquine, ribavirin, lopinavir, ritonavir, vancomycin, cefepime, and oxygen therapy	Oxygen therapy, hydroxychloroquine, azithromycin, methylprednisolone, vitamin C, zinc, and enoxaparin	-	
Complications	Diabetic ketoacidosis, pulmonary thromboembolism, OTI and mechanical ventilation, and distributive shock	NIV	NIV	NIV	-	
Outcome	Death	Resolution	Resolution	Death	Resolution	

**Article data**	**Kumaran et al.^(54)^**	**Kataria et al.^(55)^**	**AlHarmi et al.^(56)^**	**Alwaeli et al.^(57)^**		

Age	67	46	52	30		
Sex	Female	Female	Female	Male		
Past history	Laparotomy with intestinal resection for stenosis of the mesenteric artery, and secondary prophylaxis of thrombosis	-	Type 2 DM, HTN, hypothyroidism, and morbid obesity	-		
Initial symptoms	Epigastric pain, diarrhea, and vomiting	Fever, dry cough, and dyspnea	Fever, dry cough, and dyspnea	Fever, dry cough, nausea, vomiting, abdominal pain, diarrhea, and progressive dyspnea		
GIT symptoms	Yes	Yes	Yes	Yes		
Abdominal pain	Epigastric	Epigastric pain irradiating to the back	Abdominal	Epigastric pain irradiating to the back		
Pneumonia	No	Yes	Yes	Yes		
SARS	No	Yes	Yes	Yes		
Test to confirm COVID-19	RT-PCR	RT-PCR	RT-PCR	RT-PCR		
Amylase, U/L	1,483	501	47	151		
Lipase, U/L	Not done	1,541		1,022		
CPR, mg/dL	15.8	2.51	1.09	-		
Imaging test suggestive of acute pancreatitis	Abdominal CT	Abdominal CT	Abdominal CT	Abdominal CT		
Triglycerides, mg/dL	310	153	168.3	133		
Cholelithiasis or alcoholism	No	No	No	No		
Diagnosis of pancreatitis	Modified Atlanta Classification	Imaging, clinical, and laboratory	Imaging and clinical	Imaging, clinical, and laboratory		
CUM	-	-	-	-		
Medication on admission	Meropenem, metronidazole, and clyndamycin	Azithromycin, ceftriaxone, and oxygen therapy	Dexamethasone, methylprednisolone, ceftriaxone, doxycycline, azithromycin, enoxaparin, vitamin D, zinc, fluticasone, salbutamol, ipratropium, and pantoprazole	-		
Complications	NIV	NIV	NIV	OTI and mechanical ventilation		
Outcome	Resolution	Resolution	Resolution	Resolution		

**Article data**	**Alves et al.^(58)^**	**Fernandes et al.^(59)^**	**Purayil et al.^(60)^**	**Patnaik et al.^(61)^**	**Rabice et al.^(62)^**	

Age	56	36	58	29	36	
Sex	Female	Female	Male	Male	Female	
Past history		-	-	-	G4PC2 33-week gestation, obesity, pre-eclampsia, type 1 DM, and prior cholecystectomy	
Initial symptoms	Dry cough, dyspnea, malaise, and abdominal pain	Fever, headache, and dyspnea	Fever, vomiting, and epigastric pain	Diffuse abdominal pain, irradiating to the back, fever, and dyspnea	Cough and fever	
GIT symptoms	Yes	Yes	Yes	Yes	Yes	
Abdominal pain	Epigastric	Epigastric	Epigastric	Diffuse, irradiating to the back	Abdominal	
Pneumonia	Yes	Yes	Yes	Yes	Yes	
SARS	Yes	No	No		No	

**Article data**	**Alves et al.^(58)^**	**Fernandes et al.^(59)^**	**Purayil et al.^(60)^**	**Patnaik et al.^(61)^**	**Rabice et al.^(62)^**	

Test to confirm COVID-19	RT-PCR	RT-PCR	RT-PCR	RT-PCR	RT-PCR	
Amylase, U/L	544	710	249	2,861	88	
Lipase, U/L	2,993	640	>600	1,650	875	
CRP, mg/dL	-	-	29	14.6	-	
Imaging test suggestive of acute pancreatitis	Abdominal CT	Abdominal CT	No	Abdominal CT	No	
Triglycerides, mg/dL	Not done	Not done	Normal	84	210	
Cholelithiasis or alcoholism	No	No	No	No	No	
Diagnosis of pancreatitis	Imaging, clinical, and laboratory	Imaging, clinical, and laboratory	Clinical and laboratory	Imaging, clinical, and laboratory	Clinical and laboratory	
CUM	-	-	-	-	-	
Medication on admission	-	-	Azithromycin and hydroxychloroquine	Meropenem and support	Dicloxacillin	
Complications	OTI and mechanical ventilation	-	-	-	NIV and Caesarean section	
Outcome	Resolution	Resolution	Resolution	Resolution	Resolution	

**Article data**	**Bokhari et al.^(63)^**	**Samies et al.^(64)^**	**Samies et al.^(64)^**	**Samies et al.^(64)^**	**Kurihara et al.^(65)^**	

Age	32	15	11	16	55	
Sex	Male	Male	Male	Female	Male	
Past history	-	Obesity	Overweight	Prior pancreatitis	-	
Initial symptoms	Fever, sore throat, productive cough, myalgia, and diarrhea	Vomiting, epigastric pain, fever, ageusia, and anosmia	Abdominal pain, headache, chills, intermittent hematochezia, and epistaxis	Nausea and epigastric abdominal pain	Pneumonia	
GIT symptoms	Yes	Yes	Yes	Yes	? - sedated patient	
Abdominal pain	Epigastric irradiated to the back	Epigastric	Periumbilical	Epigastric irradiating to the back	? - sedated patient	
Pneumonia	Yes	Yes	Yes	No	Yes	
SARS	No		No	No	Yes	
Test to confirm COVID-19	RT-PCR	RT-PCR	RT-PCR	RT-PCR	RT-PCR	
Amylase, U/L	672	Not done	215	Not done	252	
Lipase, U/L	721	233	953	1,909	263	
CPR, mg/dL	1.58	1.47	24.11	Not done	8.53	
Imaging tests suggestive of acute pancreatitis	Abdominal CT	Abdominal CT	Abdominal CT	Abdominal ultrasound	Abdominal CT	
Triglycerides, mg/dL	150	Not done	Not done	Not done	185	
Cholelithiasis or alcoholism	No	No	No	Yes	No	
Diagnosis of pancreatitis	Imaging, clinical, and laboratory	Imaging, clinical, and laboratory	Clinical and laboratory	Clinical and laboratory	Imaging, clinical, and laboratory	
CUM	-	-	-	-	-	
Medication on admission	-	-	Piperacillin-tazobactam	-	Lopinavir-ritonavir, azithromycin, and ceftriaxone	
Complications	-	-	Acute appendicitis	-	OTI and mechanical ventilation, ECMO, and hemodialysis	
Outcome	Resolution	Resolution	Resolution	Resolution	Resolution	

**Article data**	**Bineshfar et al.^(66)^**	**Hassani et al.^(67)^**	**Bouali et al.^(68)^**	**Schepis et al.^(69)^**	**Ahmed et al.^(70)^**	

Age	14	78	60	67	47	
Sex	Male	Female	Female	Female	Male	
Past history	-	HTN and ischemic heart disease	-	-	-	
Initial symptoms	Abdominal pain, nausea, and vomiting	Positional epigastric pain, nausea, and vomiting	Respiratory failure, diffuse abdominal pain, hematemesis, and melena	Fever, epigastric pain, and vomiting	Fever, sore throat, left-sided cervical edema, fatigue, and myalgia	
GIT symptoms	Yes	Yes	Yes	Yes	Yes	
Abdominal pain	Abdominal	Positional epigastric	Diffuse	Epigastric	Diffuse/RIF	
Pneumonia	No	Yes	No	Yes	Yes	
SARS	No	Yes	No	No	No	
Test to confirm COVID-19	RT-PCR	RT-PCR	RT-PCR	RT-PCR	RT-PCR	
Amylase, U/L	1,914	1,200	-	Normal	349	
Lipase, U/L	Not done	1,450	627	Normal	>600	
CRP, mg/dL	4	-	8	Increased	2.51	
Imaging test suggestive of acute pancreatitis	Abdominal CT	Abdominal ultrasound	Abdominal CT	Abdominal CT	Abdominal CT	
Triglycerides, mg/dL	Not done	Normal	Not done	Not done	Not done	
Cholelithiasis or alcoholism	No	No	No	No	No	
Diagnosis of pancreatitis	Atlanta Classification	Imaging, clinical, and laboratory	Balthazar Classification of abdominal CT	Analysis of pancreatic pseudocyst fluid containing SARS-CoV-2 RNA	Clinical and laboratory	
CUM	-	Valsartan, clopidogrel, ASA, and atorvastatin	-	-	-	
Medication on admission	-	Remdesivir and interferon beta-1b	-	-	Hydroxychloroquine, azithromycin, and cefuroxime	
Complications	-	OTI and mechanical ventilation, kidney failure, and cardiorespiratory arrest	Laparotomy, gastrectomy, and cardiorespiratory arrest	Drainage of pancreatic pseudocyst	-	
Outcome	Resolution	Death	Death	Resolution	Resolution	

**Article data**	**Dietrich et al.^(71)^**	**Chivato et al.^(72)^**	...Continuation **Table 2.** Key dat ...Continuation **Table 2.** Key data from the case reports a from the case reports	...Continuation **Table 2.** Key data from the case reports	...Continuation **Table 2.** Key data from the case reports	

Age		72		55		
Sex		Male		Male		
Past history		Overweight and HTN		-		
Initial symptoms		Nausea and mild abdominal pain		Respiratory failure		
GIT symptoms		Yes		-		
Abdominal pain		Mild		-		
Pneumonia		Yes		Yes		
SARS		Yes		Yes		
Test to confirm COVID-19		RT-PCR		RT-PCR		
Amylase, U/L		Not done		Increased		

**Article data**		**Dietrich et al.^(71)^**		**Chivato et al.^(72)^**		

Lipase, U/L		185		Not done		
CRP, mg/dL		2.3		-		
Imaging test suggestive of acute pancreatitis		Abdominal ultrasound		Abdominal CT		
Triglycerides, mg/dL		Not done		Normal		
Cholelithiasis or alcoholism		Cholelithiasis, without cholestasis		Normal		
Diagnosis of pancreatitis		Imaging, clinical, and laboratory		Imaging and laboratorial		
CUM		Beta-blocker		-		
Medication on admission		Ceftriaxone and clarithromycin		Hydroxychloroquine, lopinavir, azithromycin, and methylprednisolone		
Complications		OTI and mechanical ventilation		Not available		
Outcome		Resolution		Resolution		

HTN: hypertension; HELLP: hemolysis, elevated liver enzymes, low platelet count; CKD: chronic kidney disease; GIT: gastrointestinal tract; SARS: severe acute respiratory syndrome; RT-PCR: reverse transcriptase polymerase chain reaction; CRP: C-reactive protein; CUM: continuous use medications; NIV: noninvasive ventilation; OTI: orotracheal intubation; ECMO: extracorporeal membrane oxygenation; DM: *diabetes mellitus*; MRI: magnetic resonance imaging; GERD: gastroesophageal reflux disease; ASA: acetylsalicylic acid; ? / - : information not available in the article.

**Table 3:** Frequencies of data from the case reports

	Mean 46.47 and median 47
Sex	22 female, 19 male, and one unknown
Comorbidities, %	47.62
Abdominal pain, %	83.33
Pneumonia, %	80.95
SARS, %	38.1
Amylase mean, U/L	779.25
Lipase, U/L	1,230.88 2
C-reactive protein mean, mg/dL	9.73
Imaging suggestive of pancreatitis, %	85.71
Diagnostic classification as per Atlanta, %	73.8 (31/42)
Complications, %*	52.38
Outcome, %*	80.95 in recovery and 11.9 deaths

* Percentage excluding articles that do not mention complications or outcome.

SARS: severe acute respiratory syndrome.

In 23 studies, including 14 cohort studies, six literature reviews, one case-control study, and two prospective studies, 12 of them concluded there is some relation between COVID-19 and the pancreas or with acute pancreatitis; seven concluded no association but considered some data, and three concluded that there is no association.

Zippi et al., performed a literature review of 14 articles that proposed five theories for pancreatic damage in COVID-19. The first one is the direct damage of the virus, which is able to bind to ACE2 receptors, also expressed in the gastrointestinal tract and in the pancreas; the second one is the increase of pancreatic enzymes by kidney failure and by the lack of elimination of enzymes by the kidneys; the third is their translocation due to altered gastrointestinal permeability; the fourth, due to the use of pancreatic toxic drugs used in the treatment of COVID-19, such as lopinavir, ritonavir, tocilizumab, and baricitinib, among others; and the fifth, due to the cytokine storm caused by SARS-CoV-2, which would attack the pancreas, causing damage to the organ.^(7)^ Along the same line, Hegyi et al., concluded that multiple organ failure that occurs in severe forms of COVID-19 resembles the lipotoxicity in severe acute pancreatitis.^(15)^ Furthermore, they suggest that early supplementation with calcium and albumin helps to reduce lipotoxicity and subsequently counteract the cytokine storm, reducing severe outcomes.^(15)^

Still regarding pathophysiology, Rasch et al., pointed out that hyperlipasemia without typical signs of acute pancreatitis is a frequent finding in patients with COVID-19, due to the impaired microcirculation in severe patients, which would explain the increase in lipase rather than an extrapulmonary finding of the viral infection.^(22)^ Goyal et al., and Rathi et al., also did not recommend associating high lipase levels with severity of pancreatic injury in COVID-19, but hyperlipasemia had a greater association with frequency of gastrointestinal signs and symptoms, a threefold increased risk for poor outcome, intensive care unit (ICU) admission, and mechanical ventilation.^(32,33)^ This relation is not fully elucidated, but Akkus et al., suggested amylase and lipase can be used as indicators of disease activity and prognosis in patients with SARS-CoV-2, and can be included in their routine follow-up.^(28)^

Wang et al., evaluated 52 patients admitted to Zhongnan Hospital of Wuhan University, from January to February 2020, and analyzed pancreatic injury as elevated lipase (>70U/L) and amylase (>90U/L). They identified a pattern of mild pancreatitis present in patients with COVID-19 pneumonia, but it may not be the result of direct viral involvement of pancreatic cells, since there was usually no positive clinical finding for acute pancreatitis.^(35)^

Contrary to the studies described, McNabb-Baltar et al., showed hyperlipasemia (>60U/L) was not associated with severe forms of COVID-19 nor with worse clinical outcomes. Furthermore, they did not associate this finding with acute pancreatitis, since 48% of patients presented with hyperlipasemia, and none had criteria - whether laboratory or tomographic - for acute pancreatitis.^(19)^

Two of the included studies diagnosed pancreatitis at autopsy examination. Lax et al., analyzed 11 bodies from patients infected with COVID-19, aged between 66 and 91 years. Acute pancreatitis was identified in four out of 11 patients, and in one third of them, no typical symptoms had been seen.^(21)^ The other study was performed by Hanley et al., with bodies from eight SARS-CoV-2 positive patients, and 25% of them had acute pancreatitis. Among them, only one patient had microscopic findings of acute pancreatitis. As a limitation, the authors of the latter study could not confirm whether the findings of acute pancreatitis were due to iatrogenesis, comorbidities, or secondary infection.^(25)^

Gubatan et al., in a cohort of eight patients, concluded that those with a history of pancreatitis were more susceptible to COVID-19; 7.8% of patients with prior pancreatitis had positive COVID-19 serology, whereas 2.8% of those had no prior pancreatitis. However, the authors did not associate an increased risk of pancreatic inflammation with SARS-CoV-2 infection, since none of the patients in the study had acute pancreatitis during viral infection.^(27)^

Idiopathic acute pancreatitis in the setting of COVID-19 has been identified in several studies. Inamdar et al., in a cohort of 32 patients infected with SARS-CoV-2 and diagnosed with acute pancreatitis by the Atlanta Classification, found the idiopathic form of pancreatitis to be the most common. Furthermore, they identified that Hispanic patients with acute pancreatitis were more likely to be diagnosed with COVID-19 than other ethnicities.^(23)^ Corroborating these findings, Szatmary et al., concluded the endocrine pancreas is more vulnerable to COVID-19 infection, and male sex, abdominal pain, metabolic stress, and tomographic findings of pancreatic and duodenal inflammation with hepatic steatosis represent a distinction of pancreatitis in SARS-CoV-2.^(26)^

Pandanaboyana et al., showed those with SARS-CoV-2 and acute pancreatitis had a significantly increased risk of developing moderate to severe or even severe acute pancreatitis, and a higher risk of secondary complications. They also had higher mortality compared to the group without the virus infection. However, this finding may have been due to the more advanced age, worse functionality score, and more severe and advanced stages of acute pancreatitis.^(31)^ Dirweesh et al., also identified higher mortality in the SARS-CoV-2 infected group. In their cohort study, they diagnosed acute pancreatitis according to the Atlanta Classification, and found a higher incidence of multiple organ failure and persistent organ failure in this cohort.^(24)^ In their prospective study, Akarsu et al., also showed that acute pancreatitis in patients with COVID-19 may deteriorate their clinical status and increase mortality.^(16)^

Not only in the adult group, Suchman et al., performed a retrospective study of patients under 18 years of age admitted to twelve New York City hospitals, between March and June 2020. Thirteen patients in the study were diagnosed with acute pancreatitis by the International Study Group of Pediatric Pancreatitis: In Search for a CuRE (INSPIRRE) criteria, ten of whom were diagnosed with idiopathic pancreatitis, with only two COVID-19 positive and the remainder negative. The authors suggest acute pancreatitis may occur in pediatric patients, and may be more common in those infected with SARS-CoV-2, particularly if there are associated gastrointestinal symptoms.^(14)^

Regarding imaging findings, Shiralkar et al., analyzed abdominal and thoracic CT and MRI scans of patients admitted with COVID-19 to the service included in the study. Ninety percent of them had pulmonary findings typical of COVID-19, and 25% of them had gastrointestinal findings of intestinal wall, pancreatitis, and cholecystitis. Among these patients, 70% had gastrointestinal symptoms upon admission, and 30% had them throughout their hospitalization. The authors call for early testing for SARS-CoV-2 in patients with typical or even atypical gastrointestinal symptoms, since it can lead to earlier diagnosis and isolation.^(30)^ Along the same line, Gupta, in a meta-analysis, suggests associating clinical-radiological tests for the diagnosis of acute pancreatitis and COVID-19 and, furthermore, warns about exposure to peritoneal fluid, if a surgical approach is necessary, since this fluid or other peritoneal fluid may contain viral particles and be a source of contamination for the staff.^(17)^

Three of the studies analyzed demonstrated no relation between the virus and acute pancreatitis. Samanta et al., in five articles analyzed in systematic review, did not conclude about this relation.^(29)^ McGuinness et al., compared hospitalization for acute abdomen, and there was no significant difference between 2019 and 2020 in severity of disease in patients with acute appendicitis (p=0.970), acute diverticulitis (p=0.333), or acute pancreatitis (p=0.803). However, this study was carried out in New Zealand, where there were few cases of COVID-19 cases during the study period.^(34)^ Miró et al., found that hospital admission for acute pancreatitis as a presentation of COVID-19 is uncommon in emergency departments, and demonstrated that in-hospital mortality does not differ between patients with acute pancreatitis, with or without concomitant viral infection. However, mortality in COVID-19 patients was higher in the setting of acute pancreatitis, perhaps due to the severity of presenting both diseases at the same time.^(20)^

Regarding published case reports, Juhász et al., pointed out that not all of them follow the case report guidelines, often skipping steps in the investigation of acute pancreatitis or not giving importance to the effect of several drugs used in the treatment of COVID-19 at hospital settings, which have also been described as a cause of acute drug-induced pancreatitis.^(18)^

The 39 case reports included in this study (some with case series) totaled 42 patients. Not all studies presented complete data of the clinical case and diagnosis of acute pancreatitis.

Most studies diverge as to diagnostic criteria, with three reports based on the Atlanta Classification; three on the modified Atlanta Classification; two on the modified Glasgow coma scale for acute pancreatitis; 17 joining clinical, laboratory, and imaging data; seven only with abdominal computed tomography (CT); two with clinical diagnosis; five with clinical and laboratory data, and one with clinical data and abdominal CT. Among the signs of acute pancreatitis on imaging, in 33 of 42 cases there were descriptions of suggestive findings, such as pancreatic edema, blurring of peripancreatic fat, necrosis, pancreatic pseudocyst, among others. Only three studies presented cases of patients with a history of mild alcoholism ^(41)^ and cholelithiasis.^(64,71)^

Among the characteristics of patients (n=42), 19 had comorbidities, sometimes combined, such as obesity or overweight (8), hypertension (10), type 1 or 2 *diabetes mellitus* (six), and hypercholesterolemia (one). The female patients appeared subtly more numerous than males - 22 and 19 cases, respectively. The mean age was 46.64 years, with a minimum of 11 years and a maximum of 78 years, with a median of 47 years and a mode of 67 years (four patients), and 36 years (four patients). Only seven studies cited the continuous use of medications, that is, beta-blocker, valsartan, clopidogrel, acetylsalicylic acid (ASA), atorvastatin, trastuzumab, losartan, metformin, insulin, omeprazole, nifedipine, carvedilol, levothyroxine, alendronate, cyanocobalamin (Vitamina B12), and alprazolam.

Among the initial symptoms that led the patient to seek medical care, gastrointestinal (64.28%), respiratory (47.61%), and fever (54.76%) problems were the most common. Regarding abdominal pain, it was typical in the epigastric region, in a band-like area, irradiating to the back (12/42); only epigastric (13/42); diffuse (8/42); in the right iliac fossa (1/42), periumbilical (1/42), or absent (3/42); four studies did not mention abdominal pain.

The diagnosis of pneumonia was made in 34 patients, 16 of whom had SARS *per se*. Only one article did not mention the diagnosis of COVID-19,^(39)^ and, in the others, it was made by reverse transcriptase polymerase chain reaction (RT-PCR).

Regarding laboratory tests, 28 studies included serum amylase results, with a mean of 779.25U/L (the highest being 3,568U/L); 32 included serum lipase, with a mean of 1,230.88U/L (the highest being 8,352U/L); C-reactive protein was cited in 25 articles, with a mean of 9.73mg/dL (the highest being 29mg/dL); and triglycerides were performed in 15 articles, with a mean of 191.54mg/dL (the highest being 310mg/dL)-nine articles did not cite the triglyceride result, but described it as normal.

The medications used during hospitalization were cited in 18 of the reports included in the study. There were antibiotics, antimalarials, antivirals, corticosteroids, anticoagulants, bronchodilators, zinc, vitamin D, pantoprazole, and ondansetrone.

Regarding the evolution of cases, the most frequent complications were the need for non-invasive ventilation (NIV) with some oxygen supplementation (11/42), followed by the need for orotracheal intubation (10/42). Other noteworthy complications were kidney failure and need for hemodialysis (5/42), use of extracorporeal membrane oxygenation (ECMO) (3/42), and cardiac arrest (3/42). Among other less frequent events in the sample were the need to conduct term (1/42) and premature (1/42) labor, diabetic ketoacidosis (1/42), pulmonary thromboembolism (1/42), refractory distributive shock (1/42), acute appendicitis concomitant with pancreatitis (1/42), pancreatic pseudocyst and its drainage (1/42), and laparotomy approach due to extensive necrosis (1/42). Two reports did not provide complications. The outcome of the cases was mostly resolution of the condition (34/42), with five deaths. Three reports did not provide the outcome.

## DISCUSSION

The pancreas as an extrapulmonary site in SARS-CoV-2 infection is still a doubt among several researchers. An interesting number of case reports suggested this association, but there is no pattern among them, which makes analysis and comparison among studies difficult. In addition, there is a small number of studies that are not case reports, but in those found in this research, most of them concluded there is some association.

The hypothesis that the increasing diagnosis of acute idiopathic pancreatitis is related to COVID-19 as a possible infectious etiology, is due to the known ability of other viruses to infect and inflame the gland.^(13)^ Therefore, associating the two causes could be plausible. Furthermore, SARS-CoV-2 causes infection in cells by binding its glycoprotein (spike protein S) to the ACE2 receptor, which is present in various tissues, including the pancreas.^(4)^ It is also known that ACE2 participates in the regulation of metabolism through its action in the gland, allowing better insulin secretion and glucose homeostasis.^(73)^ Interestingly, the expression of SARS-CoV-2 in the pancreatic tissue, in addition to supporting the hypothesis it causes acute pancreatitis, can also cause insulin-dependent *diabetes mellitus* by destroying the pancreatic islets.^(74)^ Additionally, several studies have demonstrated pancreatic involvement with histopathological analysis in autopsy of patients with COVID-19.^(9,10)^

In contrast, another pathological study that also identified viral RNA in the pancreas analyzed the SARS-CoV-2 RT-PCR Ct (RT-PCR-cycle threshold) values.^(11)^These values are used to measure the prognosis of the disease. Low values correlated with a greater risk of a serious evolution and higher mortality and, therefore, worse prognosis.^(75)^ In the study, higher Ct values were found in non-respiratory tissues, and no active viral replication or hybrid virus capture was identified in them. It was concluded that, although RT-PCR for SARS-CoV-2 was positive in non-respiratory tissues, such as the pancreas, this finding might be due to residual viral RNA in the blood of these organs. The much-feared cytokine storm and multiple organ failure, which occur in severe forms of COVID-19, resemble the lipotoxicity process in severe acute pancreatitis.^(15)^ Thus, some authors suggested that in a patient in moderate to severe state, pancreatitis may be either by systemic inflammation of COVID-19 or by the virus itself in the pancreas. Severe cases of COVID-19 are strongly related to comorbidities, mainly hypertension, followed by *diabetes mellitus*, coronary disease, and obesity, among others.^(3)^ Of the 42 patients described in the included reports, 19 presented at least one of these diseases, which may support the theory of exacerbated systemic inflammation in response to the virus that caused the pancreatic injury, and not the direct viral action in the gland.

Acute pancreatitis is a multifactorial inflammatory disease of the pancreas, and the major concern is the progression to severe forms, with high morbidity and mortality.^(76)^

The diagnosis is made using the 2012 Atlanta Classification, which includes typical abdominal pain (acute and persistent epigastric pain, of strong intensity, irradiating to the back), serum lipase or serum amylase increased by at least three times the normal limit, and imaging findings compatible with acute pancreatitis on contrast-enhanced CT, MRI, or abdominal ultrasound. The diagnosis is established with at least two of the three criteria listed, and the disease is classified as mild, moderate, or severe, according to the patient’s evolution within 48 hours. This criterion is accepted worldwide, leading to a consensus for the diagnosis of the disease, as well as better differentiation between the presentations (acute peripancreatic collection, pseudocyst, necrosis, and walled-off necrosis), and guidance for the most appropriate treatment.^(77)^

Among the case reports included in this study, only six explicitly stated that the Atlanta Classification was used, which makes it difficult to compare with the others that cited diagnosis only by imaging tests (seven studies) or clinical examination (one), because the universally established criteria were not respected, leading to hasty conclusions. Although 23 studies did not directly cite the Atlanta Classification, they respected the guideline by presenting at least two criteria, used other severity scales, such as Ranson, or modified Glasgow for acute pancreatitis, also accepted internationally. The diagnosis, in general, was well performed, with 31 studies against eight others without the Atlanta Classification. However, given the important systemic manifestations in SARS-CoV-2 infection, it is necessary to use the globally accepted criteria to properly validate the diagnosis of acute pancreatitis in a potentially severe patient. This is an important orientation, both to guide the management of disease, for statistical consideration, and for studies on viral infectivity in the pancreas, the characteristics of symptoms, and their evolution.

It is interesting to note there was a greater increase in lipase compared to amylase. Lipase is more sensitive than amylase for the diagnosis of acute pancreatitis, since it has a higher peak and stays elevated longer. However, lipase can be elevated for several reasons, such as cytotoxic effects of COVID-19 or increased intestinal permeability, as in critical illness in intense care unit (ICU), diabetes, use of opioids, and diarrhea.^(66,78)^

Several authors have noted divergences in diagnoses of acute pancreatitis, mainly because hyperlipasemia draws so much attention. Rasch et al., warned this finding is a result from impaired microcirculation rather than an extrapulmonary finding of viral infection *per se.*^(22)^ McNabb-Baltar et al., further demonstrated hyperlipasemia was not related to acute pancreatitis, since 48% of patients in the study with this finding did not present with laboratory or tomographic criteria to definitely make diagnosis.^(19)^ Along the same line, in his meta-analysis Gupta also alerted to the need of complementation of increased serum lipase with clinical and radiological findings.^(17)^ Wang et al., also reported increased pancreatic enzymes and associated this finding with pancreatic injury, but could not conclude whether there was a relation with direct viral damage to the gland.^(35)^ However, in this latter study, the classification of pancreatic injury did not follow the Atlanta Classification, with mean normal value or slightly elevated values of lipase and amylase - 77U/L and 86U/L, respectively.

Therefore, it is even more important to use the Atlanta Classification, since in the presence of SARS-CoV-2 infection, hyperlipasemia, or hyperamylasemia may occur, with or without clinical or radiological findings of acute pancreatitis. These findings should be supplemented with clinical and imaging examinations, to elucidate the diagnosis and adequately treat the patients.

On the other hand, three studies^(28,32,33)^ suggested hyperlipasemia can be used as a prognostic value in critically-ill patients with COVID-19. They demonstrated that higher values were associated with increased risk for poor outcomes, ICU admission, intubation, and mechanical ventilation time. Although McNabb-Baltar et al.,^(19)^ concluded otherwise, these other three studies have relevant numbers of patients and similar results, which help to clarify the role of increased serum lipase in these cases. Thus, dosing the enzyme, especially upon admission of critically-ill patients, may contribute to more targeted care, as a simple and low-cost test that would act as a prognostic factor. Given the serious situation in several hospitals around the world, including Brazil, at the peak of the pandemic, with ICU capacity rates above 80% in several states,^(79)^ dosing of lipase could help in the allocation of patients and predict the care they may need, such as ICU admission and mechanical ventilation, improving hospital organization and patient management.

The fact that most patients described in the included reports sought medical care for gastrointestinal symptoms (64.28%), typical or atypical of acute pancreatitis, is a worrisome fact. Most presented only with extrapulmonary symptoms, had respiratory symptoms late in their hospitalization, or presented suggestive tomographic findings by chance, such as ground-glass opacities, in the absence of respiratory symptoms. These facts have led teams to test RT-PCR for SARS-CoV-2 in these cases. Dietrich et al., described the case of a 72-year-old patient who presented with nausea and abdominal pain for 7 days. On investigation, an abdominal ultrasound showed cholelithiasis with no signs of cholestasis, and pancreatic parenchyma barely visible and apparently heterogeneous. Endoscopy and transesophageal ultrasound showed heterogeneous pancreatic tissue, with no focal masses, biliary duct with no signs of intraluminal or papillary calculi, suggesting acute non-biliary pancreatitis and no alcoholic etiology, since there was no past history of alcoholism. The patient progressively worsened until, on the fifth day of hospitalization, his chest CT scan showed bilateral ground-glass opacities. He was immediately isolated and subsequently confirmed with COVID-19.^(71)^ Thus, it is important to reinforce the use of personal protective equipment, even when treating patients without classic symptoms of the virus, since this may be an extrapulmonary manifestation of the virus or an early presentation.

The gastrointestinal tract is a known focus of SARS-CoV-2. In March 2020, Tian et al., found a 3% to 79% incidence of patients with gastrointestinal symptoms in SARS-CoV-2 infection, including anorexia (39.9% to 50.2%), diarrhea (2% to 49%), vomiting (3.6% to 66.7%), nausea (1% to 29.4%), abdominal pain (2.2% to 6%), and gastrointestinal bleeding (4% to 13.7%). Diarrhea was the most common symptom in both adults and children, and was observed before and after diagnosis. More alarmingly, adults and children could present such symptoms without manifesting any respiratory complaints.^(5)^These facts agree with those on table 2, in which most patients sought medical care without respiratory complaints, and were later diagnosed with the virus.^(6,38,41-45,48,51,54,60,64,66,67,69,71)^

The typical presentation of acute pancreatitis, with abdominal pain, nausea, and vomiting, fits into the most common set of gastrointestinal symptoms of COVID-19. Furthermore, diarrhea as an initial symptom was present in four of the reports included in this study, raising an alarm for the differential diagnosis of acute pancreatitis with SARS-CoV-2, or, in addition, alerting to the concomitant presence of these diseases, which, by causing intense systemic inflammation, increase morbidity and mortality in these patients. It is extremely important to apply the Atlanta Classification to diagnose acute pancreatitis and test for the virus, to identify these patients early, assist in their management, and protect the staff and other patients hospitalized at the same service for other causes.

Following the same reasoning, there are studies proving the presence of the virus in the peritoneal and peripancreatic fluid, and in pancreatic pseudocysts, sometimes in viral concentrations even higher than in the respiratory tract, poses a risk for infection of the staff during invasive procedures.^(17,40,54,69)^Therefore, more protective measures should be taken if the surgical management of these patients is necessary.

The measures of care for acute pancreatitis initially include aggressive volume replacement and oral fasting, and in COVID-19, depending on the status of the patient, such measures would not be taken.^(12)^ In addition, some drugs can be toxic to the pancreas and even cause drug-induced pancreatitis, such as antivirals (lopinavir and ritonavir), antipyretics, tocilizumab, and baricitinib.^(7)^ In the case reports presented on table 2, only 18 of them cited drugs used during hospitalization - among them lopinavir (5/19), ritonavir (4/19), and tocilizumab (1/19); in four patients the concomitant use of lopinavir and ritonavir was used, and in one of them the combined use of these three drugs. Early detection of patients with acute pancreatitis and SARS-CoV-2 is necessary to avoid the occurrence of drug-induced pancreatitis, although rare, in patients already susceptible to severe systemic inflammation, or worsening of an already installed acute pancreatitis.

## CONCLUSION

The literature is still divergent regarding pancreatic involvement in COVID-19. There are several confounding factors in the diagnosis of acute pancreatitis during concomitant infection by SARS-CoV-2, but the currently available information offers important warnings. First, in relation to the clinic, since in most cases the search for the health service was for abdominal pain, which should raise the suspicion of acute pancreatitis and other gastrointestinal diseases, as well as SARS-CoV-2 infection. Second, regarding the use of hyperlipasemia as a risk factor for admission to the intensive care unit, and use of mechanical ventilation. Third, the importance of following the Atlanta Classification or modified Atlanta Classification in patients with COVID-19 with increased pancreatic enzymes and/or gastrointestinal symptoms. Similarly, patients presenting with acute pancreatitis should be tested for COVID-19, since the clinical picture may be similar.

Thus, it is possible to identify early two diseases that can develop into serious and even fatal conditions, and to detect and isolate these patients more quickly. Additional studies are needed to prove the virus is capable of infecting the pancreas, but the data analyzed here are an important starting point.
